# The Effect of Cognitive Style on Individual Differences in Prismatic Adaptation: A Pilot Study

**DOI:** 10.3390/brainsci13040641

**Published:** 2023-04-08

**Authors:** Alessia Bonavita, Martina Bellagamba, Paola Verde, Maddalena Boccia, Cecilia Guariglia

**Affiliations:** 1 Department of Psychology, “Sapienza” University of Rome, Via dei Marsi, 78, 00185 Rome, Italy; 2 Cognitive and Motor Rehabilitation and Neuroimaging Unit, IRCCS Santa Lucia, Via Ardeatina 306/354, 00142 Rome, Italy; 3 Ph.D. Program in Behavioral Neuroscience, “Sapienza” University of Rome, 00185 Rome, Italy; 4 Aerospace Medicine Department, Aerospace Test Division, Pratica di Mare, AFB, 00071 Pomezia, Italy

**Keywords:** prism adaptation, after-effects, individual differences, cognitive style

## Abstract

Prism adaptation (PA) is a well-known and widely used technique for rehabilitating unilateral spatial neglect and studying sensory–motor plasticity. However, there is conflicting evidence in the literature regarding its effectiveness which may arise from differences in the type of prisms used, clinical characteristics of the patients, and the procedure used in training. Individual differences may play a role in PA effectiveness in rehabilitating neglect, affecting both its development and its effects. Field-dependent/independent cognitive style is a pervasive characteristic of individual functioning, affecting how environmental information is processed. Here, we tested the hypothesis that cognitive style plays a role in PA efficacy by submitting to a protocol of prism adaptation to 38 health participants, who were classified as field-dependent (FD, N = 19) or field-independent (FI, N = 19), by using the Embedded Figure Test. Results show that during the exposure phase, FI individuals needed a lesser number of pointing movements to reduce the deviation error than FD individuals. However, there are no differences in the extinction of sensory–motor and cognitive after-effects. These results suggest that prismatic adaptation is affected by individuals’ cognitive style since FI individuals will need fewer trials to reach adaptation and this could explain why using this rehabilitation technique with a unique, standard protocol is not always effective.

## 1. Introduction

Environmental changes or bodily evolution occur throughout life. Sensory–motor plasticity allows individuals to produce a correct motor response adapting to these changes [1,2]. Since the nineteenth century, the prism adaptation technique (PA) has been the tool of choice to experimentally study short-term sensorimotor plasticity [3] and since the late second half of the twentieth century this technique has become a well-known therapeutic tool for the recovery of unilateral spatial neglect (USN) [4], a disorder following lesions of the right hemisphere [5] of different etiologies (vascular, traumatic, oncological) and resulting in the inability to explore the contralateral space of the lesion (typically the left) in the absence of primary sensory and motor deficits [6]. The procedure consists of the use of goggles equipped with special lenses that produce a displacement of the entire visual field to the right (right optical deviation) or left (left optical deviation), resulting in lateral errors (direct effect) when the individuals wearing prisms point towards a visual target [1,7]. To correct the pointing errors and correctly perform visuomotor tasks, then, participants remap their sensory–motor spatial frame of reference; this remapping is called “recalibration”. Indeed, PA is a complex process and just wearing prismatic glasses is not enough to ensure adaptation, but the procedure requires several steps involving two different and independent mechanisms, which are recalibration and realignment [2,8]. Recalibration allows the modification of planned movements through online information derived from visual feedback, and entails an initial cognitive strategic response; the realignment consists of an automatic reorganization of the spatial maps [9]. According to Redding and Wallace [10], the recalibration would involve in addition to the spatial attention also the spatial frames of representation. A frame of reference is a set of coordinates used for coding the position of stimuli in the space; the egocentric frame of reference allows one to code the position of stimuli (for example the objects on the desk) according to the position of the observer (i.e., on the left, on the right, in front, etc.), while the allocentric frame of reference allows coding spatial positions of stimuli following coordinates independent from the position of the observer (as for example, using cardinal points) [11]. Following Redding and Wallace [10], recalibration would directly act, together with the reduction of the perceptive space, on the compromised components frameworks of spatial representation in USN, that in USN show a shifting towards the ipsilesional space [12]. Realignment allows the usage of egocentric components to recalibrate the different systems of representation producing visual, motor, and proprioceptive changes (after-effect); several studies showed that PA training based effectively reduces USN symptoms by reducing the ipsilesional bias of space representation frameworks [10,13]. Although there is much evidence about the effects of PA in healthy individuals as well as on various symptoms of USN, there is no shortage of ambiguous or negative results. Kinesthetic individual differences [14] as well as differences in procedures, participant selection criteria, research paradigms, and last but not least, the type of prisms used, represent a complex set of factors that could explain the observed variability [15,16,17]. Prisms with rightward optical deviation induce a sensorimotor after-effect, while prisms with leftward optical deviation induce both sensorimotor and cognitive after-effects [18,19,20,21]. Colent et al. [22] were the first to demonstrate that PA produces cognitive after-effect in the spatial representation of healthy individuals. In this population, the cognitive after-effect depends on the direction of the optical shift, with a pattern opposite to that observed in the USN [22,23,24] which is extended to personal and extra-personal representations and mental scales, influencing both implicit and explicit spatial tasks [1]. Furthermore, PA significantly reduces the “global process bias” [25]. The global process bias, altered in patients with temporoparietal junction lesions [26], is classically observed with stimuli in which local elements (for example small letters H) are arranged to compose an incongruent global element (for example a large letter M): individuals are faster to identify the global form of the stimulus than the local one, and show difficulty in ignoring global information when required to identify the local ones. PA thus also influences the hierarchical processing of visual stimuli [27]. 

To our knowledge, there is no systematic study on the effect of cognitive style on PA and the consequent sensory–motor and cognitive after-effects. A starting point for examining the individual variability that determines the effectiveness of PA in clinical use for the rehabilitation of neglect is to investigate the role of cognitive style, an individual pervasive characteristic that cannot be changed by learning or experience and modulates perceptual and intellectual functioning [28]. Cognitive style influences the way people process environmental information [29,30,31] and is divided into field-dependence (FD) and field-independence (FI) according to the reference frame used for encoding the information [28]. FD individuals are more susceptible to the assimilation of misleading cues preferring an external reference system to process and organize environmental information. On the contrary, FI individuals prefer an internal reference system that allows them to use cognitive restructuring mechanisms allowing them to discriminate and extract salient environmental information from the surrounding field (disembedding) and to adopt others’ perspectives in the environment (perspectivism) [28]. FD/FI is tested through tasks that require the identification of hidden figures (as in the Embedded Figures Test [32]) or by the rod-and-frame illusion, in which participants must realign a vertical line presented within a rotating external frame [33]. This study aims to verify whether FD/FI influences PA in healthy participants, having effects both on direct effect and sensorimotor and/or cognitive after-effects. To test if and how cognitive style affects PA, in the present study a group of healthy participants was submitted to a test of cognitive style and then to PA. The effect and after-effect of PA on visuo-spatial perception and visuo-motor organization were measured.

## 2. Materials and Methods

### 2.1. Participants

We recruited by word-of-mouth 38 right-handed [34] healthy individuals (20 female) among Sapienza college students, with a mean age of 25.26 years (SD = 3.252), and mean education of 15.76 years (SD = 1.979). The sample size was decided a priori in line with previous studies that investigated cognitive style [35,36]. All participants had normal or corrected-to-normal vision. Each participant underwent an anamnestic questionnaire to rule out the presence of neurological or psychiatric pathologies, and histories of alcohol or drug abuse. 

### 2.2. Assessment of Field Dependent/Independent Cognitive Style

**Figure 1:**
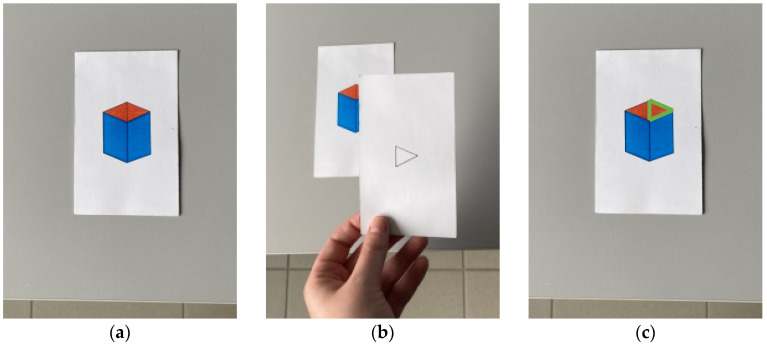
Picture and procedure of the Embedded Figures Test. The picture shows an example of the complex (**a**) and the simple (**b**) figures used and where the simple figure is hiding in the complex one (**c**).

FD/DI was analyzed using the Embedded Figures Test. The test consists of three decks of cards, two of which are formed by 12 complex figures each (version A and B of the test, respectively), and 8 showing simple ones (Figure 1). Each complex figure was shown for 15 s, and then the simple figure is placed above the complex one to cover it for 10 s. Then, the simple figure is removed, and the complex figure showed again. The experimenter, taking the time with a chronometer, asks the participant to find the simple figure within the complex one in the shortest time possible. When the participant thinks he has found it, he warns the experimenter who takes note of the time and asks to trace the contours of the figure with a stylus. If the answer is correct, the recorded response time is considered and they move on to the next item, otherwise, the experimenter continues recording the time until the correct answer is indicated or the maximum resolution time per item of 180 s is reached. The total score corresponds to the average response time of the sum of the response time on each item. 

### 2.3. Perceptual Preference Assessment

**Figure 2:**
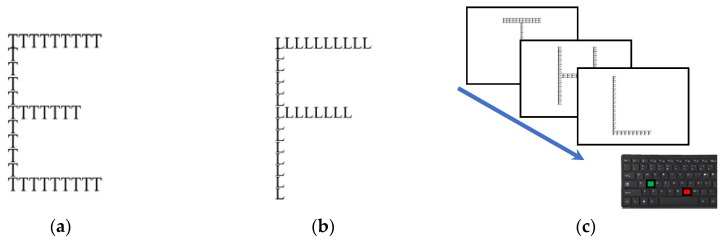
Navon task and stimuli used. The picture shows an example of stimuli (**a**,**b**) and the paradigm (**c**) used.

Global/local perception is analyzed using a computerized version of the Navon task [37]. In this version of the Navon task, all stimuli were not congruent. Indeed, the global stimuli consist of the global letter “E” or the global letter “F” made up of the letters “H”, “L”, or “T”, while the local stimuli consist of the global letters “H”, “L” or “T” made up of local letters “E” or “F”(Figure 2). Global letters measure 250 × 400 pixels (width × height) and showed in random order in the center of the computer screen. Participants must press a green response button whenever the letter “E” appears as a global or a local stimulus (target condition) and a red response button if other stimuli appear on the screen (non-target condition). The targets remain on the screen until the participant’s response. Target letter “E” appeared in 50% of trials, in half of them as a global letter and in half of them as a local one. The mean response times for identifying the target in local and in global stimuli are computed.

Navon test was used, one for the baseline assessment and the other for the after-effect assessment. In the two assessments stimuli and target were the same but the order of presentation differed.

### 2.4. Prismatic Adaptation Apparatus and Task

**Figure 3:**
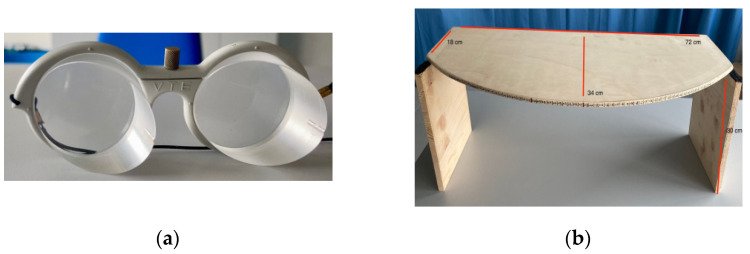
Prism adaptation apparatus. (**a**) Glasses used whit 25° lenses; (**b**) boxes used to perform prismatic adaptation.

The prismatic glasses had lenses with an optical deviation to the left of 25° (Figure 3a). A wooden prism adaptation box (30 cm high, 34 cm deep in the center, 18 cm long, and 72 cm long was built) [38,39,40]; a series of marks, corresponding to degrees, are signed on the experimenter side for allowing recording the responses (Figure 3b). The box allows participants to perform the pointing exercises while preventing them from seeing their own arm’s direction. Participants are positioned about 15 cm from the table edge with their body midline aligned with the zero point on the box. 

In each pointing trial, participants start with the right dominant hand placed in a fist on the chest at the height of the sternum and then proceed to perform a ballistic movement toward the target. The target (a pen) is placed in one of the three possible aiming positions left, right, and center in a distance that prevents tactile feedback. A total of 240 pointing trials divided between pre-adaptation, adaptation, and post-adaptation phases are delivered.

### 2.5. Procedure

#### 2.5.1. Baseline Assessment

At the beginning of the experiment, each participant completed the Embedded Figures Test and Navon tests.

#### 2.5.2. Pre-Adaptation

The pre-exposure phase includes three sets of 10 pointing each, made without the prismatic glasses: in the first set, corresponding to the baseline of the exposure phase (hereafter, visible pointing), a target was shown by the examiner placed a 10° to the right or 10° to the left of the participant’s body midline and participant moves his/her right hand in the direction of the target; in the second set (open-loop pointing) the target is shown at the central point for 5 s, after which participants have to close their eyes and then point the target position; in the third set (subjective straight ahead) participants close their eyes and point in the direction of the midline of their body. In subjective straight ahead condition, in absence of visual input the direction of pointing is computed relying on proprioceptive, motor, efference motor copy, and sensory–motor inputs; for easiness of reading thereafter we will refer to this set of inputs as “proprioceptive” [41].

#### 2.5.3. Prism Adaptation

**Figure 4:**
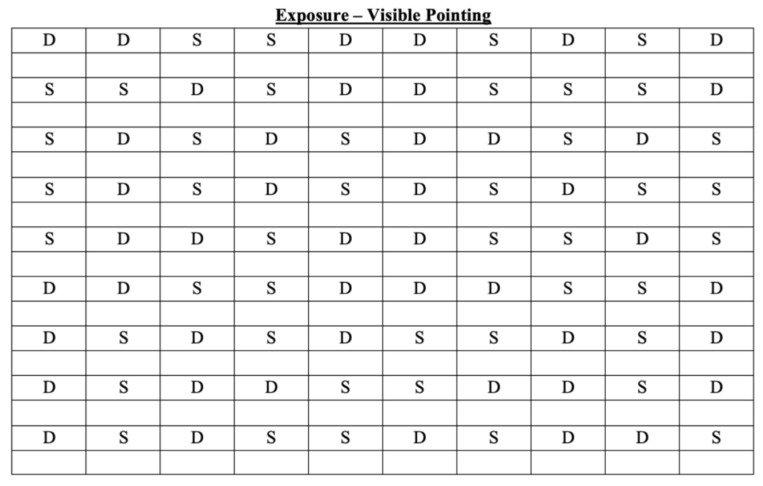
The figure shows how the 90 pointing movements are divided during the exposure phase. S = pointing movement toward the left; D = pointing movement toward the right.

The prims adaptation procedure (PA) begins when the participant wears the prismatic glasses. The target appears in one of two possible positions: the angular deviation to the left (10-degree deviation), or to the right (−10-degree deviation). Similar to Abbruzzese et al. [42], the position of the targets in the trials was randomized and counterbalanced for a total of 90 pointing movements, divided into nine sets of 10 points each, to guarantee a robust adaptation (Figure 4). The participant’s right upper limb was hidden by a wooden box covered with a curtain, leaving visible to participants their fingertips, which they can use as a reference point to calibrate movement in the direction of the target and correct the direct effect.

#### 2.5.4. Post-Adaptation

**Figure 5:**
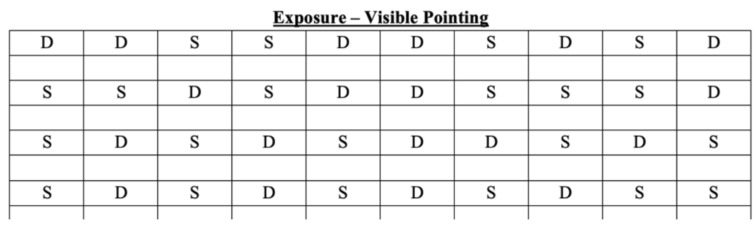
The figure shows the pointing movements performed in the post-adaptation phase. C = pointing movement toward the center.

Immediately after PA, participants repeated a series of 10 pointing movements in open-loop pointing and 10 pointing movements in subjective straight ahead conditions (Figure 5). 

#### 2.5.5. After-Effect Assessment

The Navon task was administered followed by five more series of 10 pointing movements in open-loop pointing and 10 pointing movements in subjective straight ahead conditions.

## 3. Data Analysis

Data were analyzed using the statistical program SPSS 27. Following the current methodology for FI/FD studies (see for example, [35,36,43,44]) to divide the sample size into FI and FD groups, we calculated the median value on the Embedded Figures Test, scores. Participants whose score fell above the median (median = 40.145) were classified as FD while participants whose score fell below the median as FI. With this criteria, 19 participants were classified in FD and 19 in FI groups. No significant difference was found between groups in age (t_(36)_ = −1.415, *p* = 0.166), education (t_(36)_ = −0.733, *p* = 0.468), and gender (χ^2^ _(1)_ = 1.689, *p* = 0.194). 

To verify if FI/FD affects the PA procedure during the exposure, we first calculated the mean score of the angular deviations for every one of the nine sets administered during the prism adaptation procedure when participants wear the glasses. Subsequently, we subtracted the average value of Visible pointing acquired during baseline from the average of each set of visible pointing acquired during PA, obtaining a pure timing gradient of PA (t_AP_), that was entered into our analysis. Subsequently, we performed a 9 × 2 two-way mixed ANOVA in which we entered the mean of each of the nine sets of pointing as the dependent variable (repeated measure) and the cognitive styles as the independent variable. Post hoc pairwise comparisons were corrected by using Bonferroni’s correction for multiple comparisons. 

We then tested if there were differences in FI/FD between the open-loop pointing condition and the subjective straight ahead condition registered at the baseline and in the post-adaptation phase. We performed an ANOVA with FI/FD as independent measure and open-loop pointing in the baseline and in the after-effect as repeated measure and an ANOVA with FI/FD as independent measure and subjective straight ahead in the baseline and in the after-effect as a repeated measure. We proceeded to test if FI/FD influences the extinguishment of the after-effects of PA. To obtain a pure measure of proprioceptive and sensorimotor PA after-effect, we subtracted the average value of subjective straight ahead and open-loop pointing in the baseline from the average value of each of the post-exposure series of subjective straight ahead and open-loop pointing. We performed a 6 × 2 two-way mixed ANOVA for both subjective straight ahead and open-loop pointing in which we entered the mean of each of the six sets of pointing as the dependent variable (repeated measure) and the cognitive styles as the independent variable. Post hoc pairwise comparisons were corrected by using Bonferroni’s correction for multiple comparisons. Following Boccia et al. [37], we calculated the perceptual preference index (PPI) for each participant by subtracting scores from the mean response time in local condition (${\bar{x}}_{\mathrm{ll}}$) to the mean response time in global condition (${\bar{x}}_{\mathrm{gl}}$) in the Navon test at baseline and after-effect assessment.

(1) $$PPI=\left({\bar{x}}_{\mathrm{ll}}\right)-\left({\bar{x}}_{\mathrm{gl}}\right)$$

We then performed a 2 × 2 mixed ANOVA in which we entered the pre-exposure and post-exposure PPI as the dependent variable (repeated measure) and the cognitive styles as the independent variable to test effects of FI/FD in cognitive after-effects.

## 4. Results

We verified if cognitive style affects PA. 

**Figure 6:**
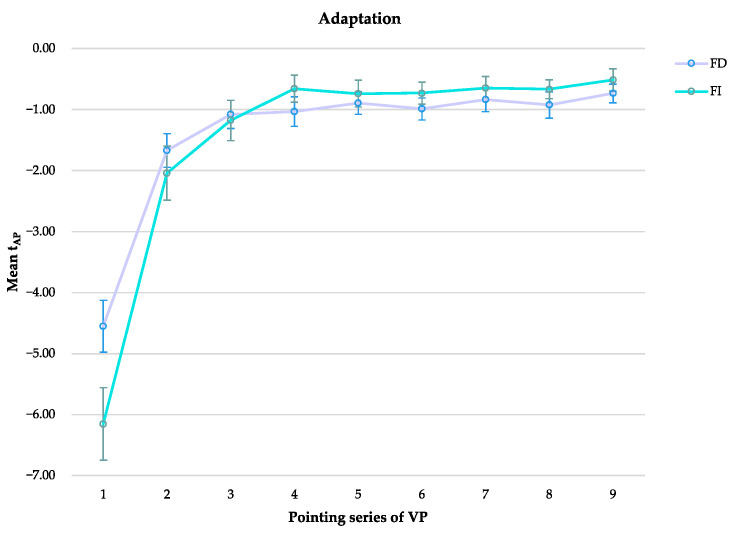
The graphic shows the time gradient of prismatic adaptation (t_AP_) means (±SE) in the nine sets of visible pointing (VP) for field dependent (FD) and field independent (FI).

Since the main effect of the timing gradient of PA significantly violated the sphericity assumption (W = 0.002, *X*^2^_(35)_ = 214.08, *p* < 0.001), we corrected the F-value with Greenhous–Geisser. All participants developed PA (F _(2.495, 89.815)_ = 123.682, *p* < 0.001, η_p_^2^ = 0.775) without a significant difference between FI and FD (F _(1)_ = 0.052, *p* = 0.822, η_p_^2^ = 0.001). We found, instead, a significant interaction between FI/FD and PA (F _(2.495)_ = 5.173, *p* < 0.004, η_p_^2^ = 0.126) (Figure 6). The pairwise comparisons show a significant difference due to cognitive style only in the first series of pointing movements (*p* = 0.035), showing in FD a greater deviation error than in FI. FI developed PA more rapidly than FD, reducing the pointing error already at the third set of pointing. The results are summarized in Table 1.

Second, we tested whether there were any differences in how FI/FD extinguished PA. 

First of all, two ANOVAs were performed to verify possible differences between pointing on the baseline and after-effect in the post-adaptation phase. The analysis concerning the open-loop pointing showed a significant effect of time (F_(1, 36)_ = 56.637, *p* < 0.001, η_p_^2^ = 0.611); no differences were present between FI and FD (F_(1)_= 3.621, *p* = 0.065, η_p_^2^ = 0.091) and in the interaction (F_(1)_ = 0.717, *p* = 0.403, η_p_^2^ = 0.020). Similar results were obtained for the subjective straight ahead condition: a significant effect of time was present for time (F_(1, 36)_= 30.986, *p* < 0.001, η_p_^2^ = 0.463), while no difference was present between FI and FD (F_(1)_ = 0.239, *p* = 0.628, η_p_^2^ = 0.007); also, the interaction did not reach the statistical significance (F_(1)_ = 1.616, *p* = 0.212, η_p_^2^ = 0.043). 

**Figure 7:**
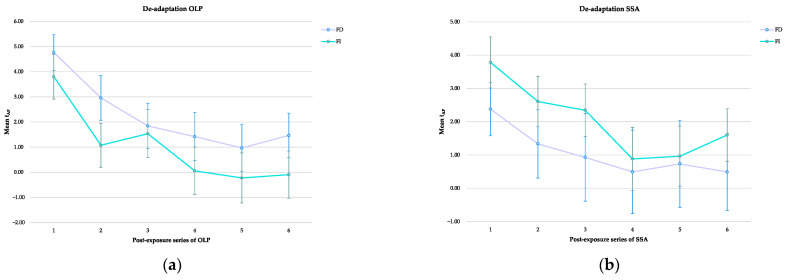
The graphic shows the time gradient of prismatic adaptation (t_PA)_ means (±SE) during the de-adaptation phase in each set of open-loop pointing (**a**) and subjective straight ahead (**b**) for field dependent (FD) and field independent (FI).

We then tested if there were differences in FI/FD during the de-adaptation. Since the sphericity of Mauchly is significantly violated for both open-loop pointing (W = 0.198, X^2^_(14)_ = 55.180, *p* < 0.001) and subjective straight ahead (W = 0.140, X^2^_(14)_ = 66.930, *p* < 0.001), F-values were corrected with Greenhous–Geisser. Data analysis shows in both open-loop pointing and subjective straight ahead a significant main effect of time (open-loop pointing: F _(2.983, 107.388)_ = 18.870, *p* < 0.001, η_p_^2^ = 0.344; subjective straight ahead: F _(2.674, 96.265)_ = 7.995, *p* < 0.001, η_p_^2^ = 0.182), but not of FI/FD (open-loop pointing: F _(1)_ = 1.147, *p* = 0.291, η_p_^2^ = 0.031; subjective straight ahead: F _(1)_ = 0.558, *p* = 0.460, η_p_^2^ = 0.015). No significant interaction with FI/FD for open-loop pointing (F _(2.983)_ = 0.667, *p* = 0.573, η_p_^2^ = 0.018) nor subjective straight ahead (F _(2.674)_ = 0.693, *p* = 0.543, η_p_^2^ = 0.019) was observed (Figure 7).

**Figure 8:**
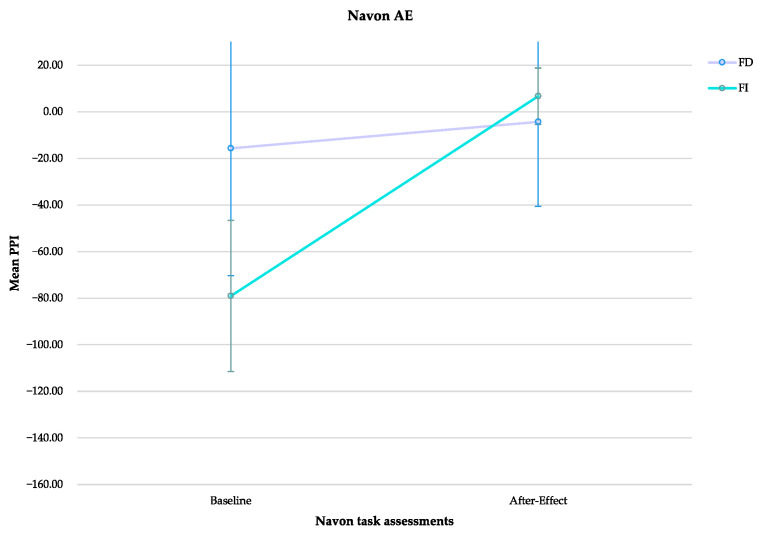
The graphic shows the perceptual preference index (PPI) scores (±SE) for field dependent (FD) and field independent (FI) in both baseline and after-effect assessments of the Navon task.

We then investigated whether FI/FD affected cognitive after-effects. There were no significant effects for cognitive after-effect (main effect of time: F _(1, 33)_ = 2.7, *p* = 0.110, η_p_^2^ = 0.076; main effect of cognitive style: F _(1)_ = 0.418, *p* = 0.522, η_p_^2^ = 0.013; interaction: F _(1)_ = 1.573, *p* = 0.219, η_p_^2^ = 0.046) (Figure 8).

## 5. Discussion

PA is a well-known, long-used technique that in the last decades has been successfully applied to the rehabilitation of USN [4,45].

The literature demonstrates that healthy individuals wearing prisms show an error in pointing to visual targets corresponding to a displacement coherent with the displacement of the visual scene induced by prisms. This error is rapidly recovered due to a sensory–motor reorganization in successive trials (adaptation phase) but reappears after the prisms are removed as an after-effect, consisting of a displacement in pointing with a direction opposite to that observed during adaptation. In USN the after-effect allows patients to catch stimuli and to act in the contra-lesional, neglected hemispace and several studies demonstrated that administering PA training may result in recovery of USN [46]. However, different studies found different degrees of PA effect in healthy individuals and of effectiveness in rehabilitating USN patients. This may be due to differences in the setting or the type of prisms; in studies about efficacy in rehabilitating USN some other variables may occur, such as the severity of USN, presence of other neuropsychological disorders that may interfere with the recovery, distance from the onset, and number and length of PA sessions. Another variable that may underlie the differences in effect size in different studies may consist of individual differences in the susceptibility to the effects and the after-effects of PA.

In this preliminary report about the effect of PA on proprioceptive, sensory–motor, and cognitive after-effects in healthy subjects, we tested for the very first time the possible role of cognitive style in prismatic adaptation. Our results show that FD/FI impacts the way in which PA occurs, being the adaptation to prisms faster in FI individuals, who also showed smaller deviation during pointing as an effect of prisms. One possible explanation of this difference refers to how FD/FI perceive space. The recalibration and realignment strategies that allow PA rely on egocentric and allocentric spatial representations [10]. Updating the displaced egocentric representation based on the object position is an automatic process involving the calculation of the amplitude of the angular displacements in which the body orientation axis becomes the reference point with which the egocentric coordinates update [47]. When FD individuals change their own perspective, they align their internal frame of reference with the environment. This process is time-consuming and more prone to mistakes. Instead, when FI individuals change perspective they could easily update their frame of reference using the cognitive restructuring processes that characterize their cognitive style [48]. 

A greater amplitude of low-frequency fluctuations in the frontoparietal networks could be associated with the disembedding process engaged by FI, and the activity in the medial prefrontal cortex is associated with more effective cognitive inhibition of information deriving from the field [49]. Despite conflicting results on brain areas engaged during PA, the parietal cortex and the cerebellum appear to play a key role. Some authors find that the parietal cortex is engaged during the recalibration phase and that it modulates the cerebellum-coordinated realignment phase [50,51]; others argue that there is an activation of the posterior parietal areas (particularly the parietal cortex) and cerebellum in all phases of PA [8,50,52]. 

To attain the effects of PA on the Navon task, previous studies showed that PA significantly reduces the local bias in brain-damaged patients with lesions involving the tempo-parietal junction [26], demonstrating that PA may modify also non-spatially lateralized deficits of USN. In addition, Bultitude and Woods [27] found that PA reduces the global bias in healthy individuals. We did not observe any of these effects, perhaps due to the fact that the Navon task we adopted is significantly different from those used in previous studies. In the present study, indeed, a target letter “E” and a non-target letter “F” were presented as a global stimulus formed by one of three different letters (“H”, “L”, “T”) or as a local stimulus to form one of three different letters (“H”, “L”, “T”). At variance, Bultitude et al. [26] and Bultitude and Woods [27] used a more classical Navon task, in which the target letter appears as a local stimulus forming the global target or the global non-target stimuli and the local non-target stimulus appears as a local stimulus forming the global target or the global non-target stimuli. In this case, half of the stimuli were congruent (that is global target is formed by local targets and the non-global target is formed by local non-targets) or not-congruent (that is global target is formed by local non-targets and global non-target by local targets). It is possible to hypothesize that this second type of Navon task requires more cognitive resources than the type we used and that in presence of a more difficult task, the PA effects and after-effects became more evident.

In other words, we suggest that PA affects the processing of incongruent/congruent stimuli, more than it affects a simple detection task.

The cognitive style seems not to affect the extinction of PA, however, although no significant effects were found at proprioceptive, sensorimotor, and cognitive levels, data show a trend in FI individuals to extinguish PA earlier than FD individuals, at least in open-loop pointing condition. A possible explanation could be due to methodological errors. Indeed, all the individuals perform the alternative version of the Navon task immediately after the PA. This time window is crucial to determine if the after-effects had occurred and at what strength. Some studies have pointed out that after-effects start to decrease within 15/20 min after PA [53,54,55]. We did not take note of how much time each participant took to perform the Navon task immediately after PA; thus, we may assume that our results could have been affected by the temporal discrepancy that occurred between when each experimental subject completed again the Navon before performing the last series of pointing movements. Thus, it is possible that differences between FI and FD depend on the fact that FD needed more time for performing the cognitive tasks (Navon) and started the after-effect assessment later than FI. Were this the case, since FD performed this assessment at a distance from PA superior to that at which the same assessment was performed by FI. 

A possible limitation of this study could be that the relatively small sample we collected is not large enough to evidence differences in cognitive style. Indeed, a larger sample would allow us to divide the sample in tertials according to their performance on the Embedded Figures Test, and to exclude the central tertial form analyses, allowing us to better contrast FD and FI. 

However, even if results do not show striking differences between FI and FD, this pilot study suggests that cognitive style can play a role in PA, especially in the timing of adaptation and recalibration, and suggest further studies to better analyze the role of cognitive style on PA.

## 6. Conclusions

In conclusion, this pilot study, even if not adding anything about the understanding of the underlining mechanisms of PA, shows that cognitive style plays a role in how PA takes place. This influence could explain on the one hand how stable functioning dimensions influence sensorimotor plasticity and on the other hand, suggest that FI/FD cognitive style may play a role when PA is applied in a clinical context for example, affecting the efficacy of PA training in USN rehabilitation. Namely, the present result suggests the need for further studies for evaluating if PA training reaches the same level of efficacy in FI and FD patients. Despite the encouraging preliminary results, in further investigation, the experimental procedure needs to be revised based on the problems that emerged in this pilot study.

## Figures and Tables

**Table 1:** The table summarizes descriptive statistics of the time gradient of prismatic adaptation (t_PA_) by cognitive style (CS) and pointing series for the visible pointing condition. FD = Field dependent; FI = Field indipendent.

CS	Pointing Series	Mean	Standard Error	Confidence Interval 95%	
				*Lower limit*	*Upper limit*
FD	1	−4.553	0.516	−5.599	−3.506
	2	−1.668	0.369	−2.417	−0.92
	3	−1.079	0.283	−1.654	−0.504
	4	−1.032	0.231	−1.5	−0.563
	5	−0.895	0.203	−1.306	−0.484
	6	−0.989	0.18	−1.354	−0.625
	7	−0.837	0.191	−1.224	−0.45
	8	−0.926	0.186	−1.304	−0.548
	9	−0.737	0.17	−1.082	−0.392
FI	1	−6.153	0.516	−7.199	−5.106
	2	−2.042	0.369	−2.79	−1.294
	3	−1.179	0.283	−1.754	−0.604
	4	−0.658	0.231	−1.126	−0.19
	5	−0.742	0.203	−1.153	−0.331
	6	−0.732	0.18	−1.096	−0.367
	7	−0.647	0.191	−1.034	−0.26
	8	−0.668	0.186	−1.047	−0.29
	9	−0.516	0.17	−0.861	−0.171

## Data Availability

Data are available from the corresponding author upon request.
